# Laser Powder Bed Fusion of Fe-10 at% Ni and Fe-10 at% Si Soft-Magnetic Materials from Powder Blends

**DOI:** 10.3390/ma18194471

**Published:** 2025-09-25

**Authors:** Jan-Simeon Ludger Bernsmann, Paul Stahl, Luca Christian Matzel, Johannes Henrich Schleifenbaum

**Affiliations:** Digital Additive Production (DAP), RWTH Aachen University, 52074 Aachen, Germany

**Keywords:** Laser Powder Bed Fusion (PBF-LB/M), additive manufacturing, soft-magnetic materials, powder blends, AI-based process optimization

## Abstract

Soft-magnetic materials can benefit significantly from additive manufacturing using Laser Powder Bed Fusion of metals with laser beam, as this technology allows the production of parts with complex geometries. In this study, two iron-based alloys were investigated: Fe-10%Ni (at%) and Fe-10%Si (at%), which are known for their promising soft-magnetic properties. A parameter study was first conducted to optimize the process settings with the goal of maximizing the relative density, which strongly influences magnetic performance. Using AI-based optimization software (xT-Saam by Exponential Technologies Ltd., Riga, Latvia), geometrically simple specimens with a relative density of ≥99.95% were successfully produced. Utilizing the developed parameter sets, toroids were manufactured and heat-treated to improve their magnetic properties. The best obtained ferromagnetic properties were H_C_ = 1621 A/m (coercivity) and µ_R_ = 305 (permeability) for Fe-10%Ni, and H_C_ = 300 A/m and µ_R_ = 1114 for Fe-10%Si.

## 1. Introduction

Due to the global goal of reducing CO_2_ emissions, the use of environmentally friendly energy technologies, such as electric vehicles, is increasing [1,2,3,4,5,6,7,8,9,10]. Therefore, highly efficient electrical machines, e.g., transformers or motors, are needed [11]. Within these components, soft-magnetic materials, characterized by low coercivity, high permeability, and minimal hysteresis losses, are used. Traditional manufacturing routes for Fe-Ni and Fe-Si alloys often involve cost-intensive melting, forging, and rolling steps to achieve the required microstructure and magnetic performance. To further improve efficiency, lightweight design methods or tailored soft magnets are used to make these components more compact while maintaining performance [12,13,14,15,16,17,18,19,20,21,22,23,24]. Through the usage of additive manufacturing (AM), it is possible to economically produce a complex geometry without the need for tools, in contrast to conventional manufacturing processes [13,16,20,24,25,26,27,28,29,30,31,32,33,34,35]. This allows manufacturing with less material waste and lower CO_2_ emissions compared to conventional manufacturing methods, for example, the powder metallurgy route, since unused material can be reused or recycled, and no material waste is generated by machining [17,36,37,38,39,40]. Due to high material prices, this is relevant for materials such as rare earths and also for iron alloys with cobalt, nickel, or silicon, which are commonly used for soft magnets [12,21]. Laser Powder Bed Fusion with a laser beam of metals (PBF-LB/M) is versatile to change the chemical composition of the material to be processed. If the chemical components are available in pure and powder form and morphology, they can be blended prior to the process and then administered in the process, instead of using pre-alloyed powder [11,41,42]. PBF-LB/M is one of the established methods for processing these blended materials and was, therefore, chosen for the present work to additively produce soft magnets from iron–nickel (Fe-Ni) and iron–silicone (Fe-Si) powder blends [12,31]. This approach promises lower material costs and easier tailoring of alloy chemistries but poses challenges for achieving complete mixing, evaporation of volatile elements (e.g., Si), and control of local melting dynamics. Nevertheless, the fabrication of soft magnets by PBF-LB/M is challenging, since the microstructure not only shows a directional anisotropy but also leads to exceedingly small grains due to the small melting baths compared to conventional fabrication methods, which has effects on the magnetic properties [43,44,45,46,47,48,49]. PBF-LB/M has already been used in past studies to process Fe-Ni and Fe-Si alloys, but with varying degrees of Ni and Si content [21,50]. The Ni content of Fe-Ni alloys is usually 30–80 at% [21,32,51,52]; however, these alloys possess their maximum magnetizability at 10–15 at% Ni content [52,53]. As for Fe-Si alloys, 6.5 wt% is a typical Si content since the material becomes brittle and cracks occur during processing at higher Si content [54,55]. Therefore, the Si content in this work was reduced to 5.29 wt%. to produce crack-free parts, which corresponds to an atomic fraction of 10%. For these reasons, Fe-10%Ni and Fe-10%Si were used as soft-magnetic materials in this work. Additive manufacturing by PBF-LB/M has been extensively applied to pre-alloyed soft-magnetic materials, achieving relative densities ≥ 99.9% and coercivities below 500 A/m through careful adjustment of processing parameters and powder bed pre-heating [55,56]. These studies demonstrate that temperature management and powder dynamics are critical to obtaining a homogeneous microstructure and minimizing residual stresses. In contrast, in situ alloying via powder blends—in which two or more elemental powders are mixed during the build process—has been investigated only sporadically. Early work on Ni-based powder blends reported overall densities of about 98% and suboptimal magnetic performance, which were attributed to incomplete mixing reactions and local overheating phenomena [57,58]. Meanwhile, AI-assisted parameter optimization in additive manufacturing is rapidly gaining traction as a systematic means of tackling complex, multivariate problems [59,60,61].

The alloys were selected due to their exceptionally low coercivity and favorable frequency-dependent losses reported in previous studies. In contrast, Fe-10 at% Si offers high relative permeability and reduced eddy current losses, making it attractive for high-frequency magnetic components. By comparing these two alloys processed via PBF-LB/M from powder blends, we aim to elucidate the trade-offs between coercivity and permeability in additively manufactured soft-magnetic materials. Suitable process parameters were evaluated for these alloys, and the relative (rel.) density was maximized as a target parameter in order to achieve adequate magnetic material behavior [21,54,62]. Fe-10%Ni and Fe-10%Si were intentionally chosen as soft-magnetic materials to explore alloys with optimal magnetic performance, which had not been extensively investigated in previous additive manufacturing (AM) studies. A novel AI-based software (xT-Saam (https://www.x-t.ai/xt-saam/) by Exponential Technologies Ltd.) was applied to systematically optimize process parameters, achieving extremely high rel. densities (≥99.95%), far surpassing conventional manual or empirical optimization methods. Utilizing these optimized parameters, toroidal specimens were manufactured to systematically quantify the magnetic properties. Additionally, the combined methodological approach of AM and subsequent heat treatment (HT) cycles, according to the established reference literature, was employed to further enhance magnetic performance [45,55]. Despite the demonstrated performance of pre-alloyed powders in PBF-LB/M, their high cost and limited material flexibility motivate the exploration of in situ alloying via powder blends. However, the process–structure–property relationships for Fe-10%Ni and Fe-10%Si blends remain poorly understood, and no study has yet leveraged AI-driven methods to systematically map the achievable density and magnetic performance space. This gap hinders the adoption of cost-effective powder-blend strategies in industrial soft-magnetic applications. Subsequently, toroids were manufactured using the developed processing parameters (AI-driven parameter optimization workflow (xT-Saam)), and magnetic properties were validated and critically compared with the established literature values, thus providing valuable benchmark data and extending the current understanding of AM-processed soft-magnetic materials [45,63].

## 2. Materials and Methods

### 2.1. Powder Materials (Blends)

**Figure 1:**
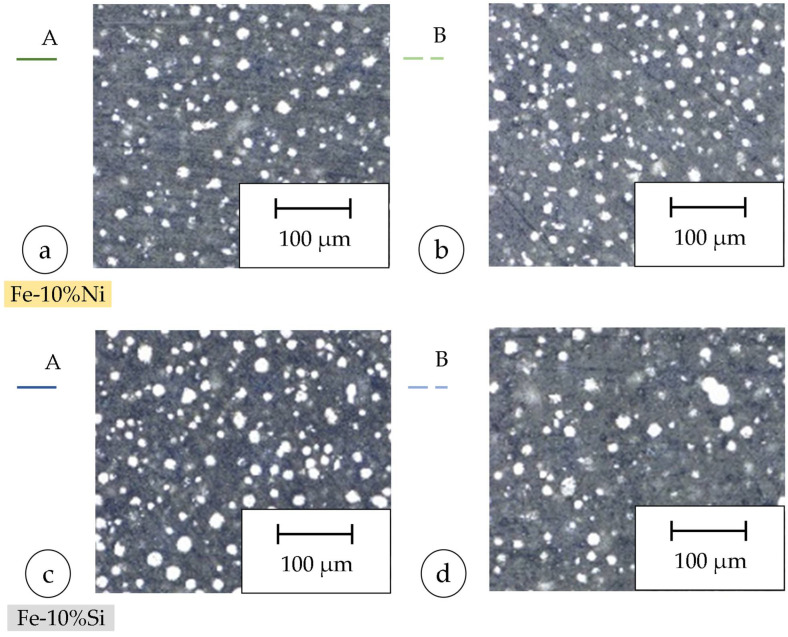
Reflected light microscope images: cross-sections of (**a**,**b**) Fe-10%Ni and (**c**,**d**) Fe-10%Si powders—left side, Batch A; right side, Batch B.

Elemental metal powders of iron (Fe), nickel (Ni), and silicon (Si) were used, with particle size distributions (PSDs) of <45 µm for Fe and 10–45 µm for both Ni and Si, as specified by the manufacturers. Powder blends with a chemical composition of 90 at% Fe and 10 at% Ni or Si were prepared by mechanical mixing. Additionally, cross-sections of the embedded powder samples were examined by reflected light microscopy to assess particle morphology and detect potential porosity (cf. Figure 1a–d).

**Figure 2:**
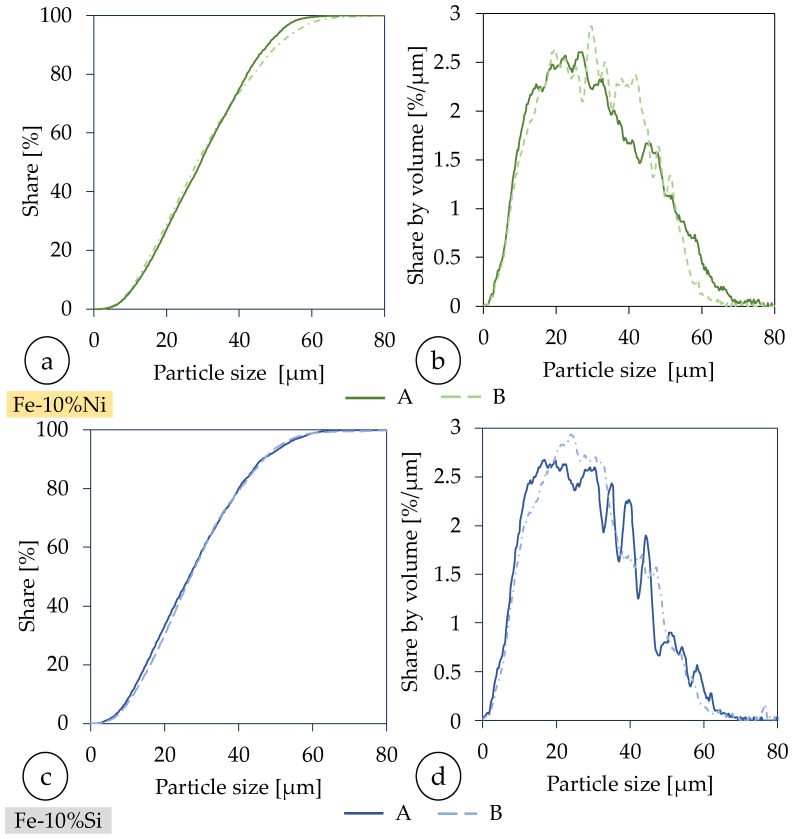
PSD measurement of Fe-10%Ni: (**a**) cumulative distribution, (**b**) frequency distribution; Fe-10%Si: (**c**) cumulative distribution, (**d**) frequency distribution.

Particle size distribution was analyzed using a Camsizer X2 (Microtrac Retsch GmbH, Haan, Germany). Two separate batches (A and B) were prepared for each blend: batch A was used for process parameter development, and batch B for the fabrication of toroidal components, in order to eliminate potential effects from sieving or powder recycling (cf. Figure 2a,b and Figure 2c,d).

### 2.2. PBF-LB/M-System

An AconityMini (Aconity3D GmbH, Herzogenrath, Germany) was used as the PBF-LB/M system for the experiments in this work. This system is equipped with a laser from the company nLIGHT. It has a wavelength of Lamda = 1070 nm, a maximum output power of P_L_ = 400 W, and a spot size of d_S_ = 90 µm. The PBF-LB/M process took place under a protective gas atmosphere of argon with a protective gas volume flow of V_Ar_ = 1.2 L/min. The oxygen (O_2_) content at the beginning of the PBF-LB/M process was <1000 parts per million (ppm) O_2_ and was constantly sinking to 0–1 ppm.

### 2.3. Reflective Light Microscopy (Relative Density) and Chemical Composition Analysis

A VHX-6000 digital microscope (Keyence GmbH, Frankfurt am Main, Germany) was used to examine the samples for rel. density analysis. This allowed the acquisition of panoramic images and the measurement of the rel. density and porosity of the powder samples. For this purpose, the specimens were embedded in Bakelite in a compression mounting press, type Qpol50-2 (QATM GmbH, Mammelzen, Germany). Subsequently, the preparation was conducted over several grinding and polishing stages. The rel. density of the specimen was measured after preparation using five images. The robustness of the measurement results increased with the uniform distribution of the measurement areas over the surface of the specimen. The evaluation software of the microscope was based on a black/white comparison. White pixels in the image represent the completely remelted material, and black pixels the pores or defects.

**Figure 3:**
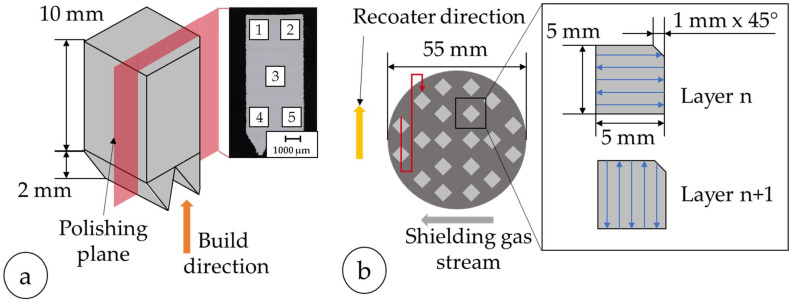
Arrangement of the samples on the build plate with order in which they were built (red arrow), and dimensions and scan strategy (blue arrows represent the scan vectors).

Accordingly, the rel. density of the measurement areas was calculated from the ratio of the number of black pixels to the number of all pixels. To calculate the rel. density of the sample, the average value was formed from the five measuring points 1–5, approx 0.9 mm apart from the edges, and one measurement in the middle of the specimen (cf. Figure 3a).

Elemental analyses of the samples were performed using the Bruker CTX portable X-ray fluorescence spectrometer (XRF) (Bruker, Billerica, MA, USA).

### 2.4. Parameter Study

Because of the interactions between the process parameters and the target parameter, rel. density, a linear optimization was not possible. Therefore, a parameter study was conducted, which was an iterative approach to develop stable processing parameters that resulted in a rel. density of ≥99.95%. The samples shown in Figure 3a) (approx. 5 × 5 × 10 mm^3^) were used for this purpose. The teeth-like structure on the bottom allowed for easy detachment of the samples from the build plate.

One side featured a chamfer whose depth was equal to the polishing plane where the rel. density was measured. This guarantees that this plane was the same for all the samples. The scan strategy was alternating with a rotation angle of 90° to reduce the anisotropy in these cuboid samples [64,65,66] (cf. Figure 3b). A steel (1.0037) substrate plate was used. A coater with a rubber lip applied the powder to the substrate plate. The inert gas flow was perpendicular to the recoater direction (cf. Figure 3b) [67]. 

The varied process parameters were the laser power P_L_, scan velocity v_S_, and hatch distance Δy_S_. The layer thickness was set to D_S_ = 30 µm (cf. Table 1).

The process window to be investigated was determined based on a literature review based (cf. Table A2) on the described correlation of the magnetic properties and the target parameter. Since there were interactions between the setting parameters regarding the rel. density, no linear optimization could be performed [68].

No pre-heating of the build plate was applied in this study to focus exclusively on the influence of laser parameters on powder blend melting and mixing. Pre-alloyed powders were also not used, as our goal was to assess the viability of in situ alloying via powder blends.

### 2.5. Experimental Design, Data Collection, and Regression

The experimental procedure involved multiple iterations for each investigated material (Fe-10%Ni and Fe-10%Si) to maximize the rel. density, a critical parameter for the magnetic properties. PBF-LB/M parameters such as laser power, scan speed, hatch distance, and layer thickness were systematically varied [68,69]. For efficient exploration of the parameter window and optimization of rel. density, the AI-based optimization software xT-SAAM (Exponential Technologies Ltd., Latvia) for Simulation Analysis and Modelling was employed. This software utilizes Advanced Space Filling Initialization to distribute experimental points evenly across the parameter window. After initial experiments, the software assigns probabilities to regions within the process window, prioritizing parameter combinations based on proximity to desired outcomes. Iterative optimization continued, guided by a genetic algorithm, which applied principles of natural selection and inheritance to refine parameter combinations progressively, enabling targeted exploration of optimal process windows. Regression modeling within xT-SAAM relied on Random Forest Regression, well-suited to address nonlinear relationships, interactions among parameters, and outliers. Measurement data were divided into training, creation, and test datasets. Using Cross-Validation (CV), 100 distinct models were generated, each tested against independent data subsets. An ensemble algorithm combined these models, enhancing predictive accuracy. Model performance was assessed through CV-R^2^ metrics, with CV-R^2^ ≥ 0.5 indicating satisfactory predictive capability. Logarithmic transformation of porosity data was applied due to the narrow range of the achieved rel. densities (95–100%), which improved model accuracy by evenly distributing data across the scale. Additionally, the Gini value analysis within the software identified the relative influence of each independent parameter on the rel. density, guiding further experimental iterations [70].

Iterations proceeded until the predefined rel. density target (≥99.95%) was consistently achieved for both materials, confirming the effectiveness and robustness of the AI-driven experimental design and regression approach.

### 2.6. Magnetic Properties

**Figure 4:**
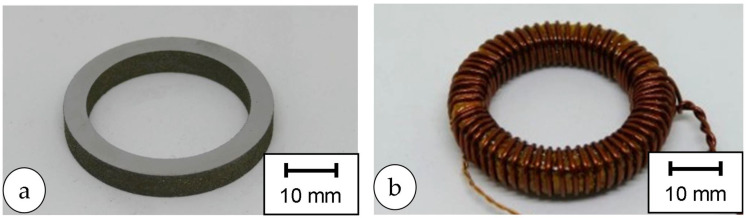
Addtive manufactured Fe-10%Ni Toroids: (**a**) before and (**b**) after coiling with copper for the SST analysis.

Toroids with an inner and outer diameter of 35 mm and 45 mm and a height of 5 mm were manufactured to measure the magnetic properties (cf. Figure 4a). The toroids were coiled with copper wire for this purpose (cf. Figure 4b).

Since both the parameter study samples and the toroids were produced from solid, nearly identical thicknesses, the results from the parameter study could be applied to the toroids. To minimize directional dependence in the magnetic properties (anisotropy), the scan strategy for the toroids was adjusted: instead of using a 90° rotation between layers—as was performed for the cuboids—a rotation angle of 67° was chosen. This non-orthogonal scanning approach helps to distribute the thermal effects more evenly during the build process, leading to a more isotropic microstructure [64,65,71,72,73,74]. The magnetic properties (magnetizability and iron losses) of the materials were measured using a permagraph Electrical (single sheet) Steel Tester (SST) MPG 200 (Brockhaus Messtechnik GmbH & Co. KG, Lüdenscheid, Germany).

The coercivity H_C_ and the relative permeability µ_R_ were measured at a frequency from f = 20 Hz up to 3000 Hz and a polarization of J = 0.1 T up to 1.8 T. The magnetization and polarization ranges are shown in Table 2.

Due to the limited output voltages and currents of the measurement system, a polarization of J = 1.8 T could not be achieved for all the frequencies. The measurements were then performed in each case up to the highest technically feasible magnetic excitation.

### 2.7. Dynamic Differential Scanning Calorimetry

Dynamic differential scanning calorimetry (DSC) measurements were performed using STA 449 F3 Jupiter (NETZSCH-Gerätebau GmbH, Selb, Germany). The device allows simultaneous recording of DSC and thermogravimetric (TG) signals under identical experimental conditions. The samples were placed in standardized crucibles and heated at a rate of 10 K/min in a controlled argon atmosphere. Gas flow rates were regulated using integrated mass flow controllers to ensure reproducible conditions. Thermal events such as melting, crystallization, and glass transitions were detected by a heat flow DSC sensor. Data acquisition and analysis were carried out using the Proteus^®^ (Version 1.1) software, applying automatic baseline corrections and peak evaluations. The system’s vacuum-tight design minimized measurement drift and allowed for precise control of the sample environment.

### 2.8. Heat Treatment

Based on literature review and similar alloys, multiple HT cycles were used [46,75,76,77]. The differing effects of heat treatment on Fe-Si and Fe-Ni soft-magnetic materials can be attributed to their fundamentally different magnetocrystalline anisotropy, phase behaviors, and sensitivities to internal stresses. Fe-Ni alloys, characterized by low anisotropy and high magnetoelastic sensitivity, respond more significantly to stress relief and structural relaxation during annealing. In contrast, Fe-Si alloys—particularly those with higher silicon content—exhibit stronger textural influences and reduced mobility of defects, making their magnetic properties less sensitive to moderate heat treatments. These intrinsic differences necessitate tailored thermal processing strategies for each alloy system [55,78,79]. For consistent and precise reference in the subsequent discussion, abbreviated sample codes have been assigned to each combination of chemical composition and heat treatment condition, as outlined A_xy_-C_xy_. These codes will be used throughout the manuscript to refer to the respective specimens. To reduce the internal stresses, the Fe-10%Ni samples were held for 5 h at 340 °C in a furnace (KLS 45/13, Thermconcept GmbH, Bremen, Germany), while the Fe-10%Si samples were held for 5 h at 700 °C. These samples were labeled as Bxx. The independent HTs are summarized in Table 3.

The Fe-10%Ni samples were held for 1 h at 1200 °C, while the Fe-10%Si samples were held for 1 h at 1100 °C. These are the samples Cxx. The oven was turned off afterwards, and the samples were left to cool down to ambient temperature in the oven. The heating rate was 5 °C/min. To avoid possible influences by oxidation, the heat treatment was carried out under a protective gas supply with argon [67]. The temperature was adjusted to 1100 °C after initial ring core samples made from Fe-10s%Si were destroyed during heat treatment at 1200 °C due to excessive oxidation. This oxidation was caused by an uneven protective gas atmosphere inside the furnace, leading to instability during high-temperature exposure. The untreated toroids (Axx) were used as a reference to the heat-treated ones.

## 3. Results and Discussion

### 3.1. Powder Materials (Blending)

Powder blends with 90 at% Fe and 10 at% Ni and Si, respectively, were created by mixing elemental powders. These manufacturers’ specifications regarding the PSD of the used elemental powders are shown in Table 4 [34,80].

The PSD of the three investigated materials shows notable differences. The iron powder exhibits a relatively broad distribution with particle sizes below 45 µm, indicating a wide range of particle sizes. In contrast, both the nickel and silicon powders have a narrower PSD, predominantly between 10 and 45 µm. This suggests that the nickel and silicon powders are more uniformly sized compared to the iron powder. The differences in PSD are important as they can significantly influence powder flowability, packing density, and overall process stability in additive manufacturing applications. The metal powder blends used in this study were characterized with regard to their particle size distribution using a Camsizer X2 particle size analyzer (Microtrac Retsch GmbH, Haan, Germany). The characterizing values of the powder size distribution are summarized in Table 5. The given Dxx values indicate the portion of the powder particles and their distribution in the measured interval. For example, for the Fe-10%Ni powder (Batch A), 90% of all the measured particles were smaller than 47.92 µm.

A parameter study was conducted as an iterative approach to develop usable processing parameters which result in a rel. density of ≥99.95%. Two batches were used for the parameter study (A) and the manufacturing of the toroids (B). Batch A consisted of a freshly prepared powder blend, whereas Batch B comprised the residual powder from Batch A, topped up with fresh powders to maintain the required quantity. The reflected light microscope (RLM) images of the cross-sectioned, embedded powder are shown in Figure 1. The shape of the powder particles is, for the most part, spherical, which results in higher packing densities of the powder bed compared to other shapes [81]. Furthermore, no porosity can be detected within the powder particles.

The PSD of the two batches, A and B, Fe-10%Ni and Fe-10%Si, is shown in Figure 2. Since the PSD of the freshly prepared powder blends B_Ni_ and B_Si_ differ only slightly for both materials, the transferability of the results of the parameter study to the manufacturing of the toroids is ensured. The cumulative distribution Q(x) is shown in Figure 2a. Figure 2b shows the frequency distribution q(x) of the particle sizes for the Fe-10%Ni batches and analog to the Fe-10%Si accordingly in Figure 2c,d. The cumulative distribution indicates the fraction of all the particles with a particle size less than or equal to a fixed value and, therefore, results from the integration of the frequency distribution. The particle size distribution shifts toward coarser fractions from Batch A to Batch B as powder agglomerates form during PBF-LB/M, and fine particles are removed by the process gas stream and captured in the system filter.

### 3.2. Relative Density and Chemical Composition

In Table 6, the process parameters that led to the highest rel. densities for both Fe-10%Ni and Fe-10%Si alloys during the parameter study are presented. For Fe-10%Ni, sample number 4 from the third iteration achieved a rel. density of 99.960% using a laser power (P_L_) of 180 W, scan speed (v_s_) of 600 mm/s, and hatch distance (Δy_S_) of 60 µm. In the case of Fe-10%Si, sample number 28 from the first iteration reached an even higher rel. density of 99.974% with a laser power of 140 W, scan speed of 550 mm/s, and the same hatch distance of 60 µm. These parameter sets were used as the basis for further investigations, including the production of crack-free toroidal specimens.

**Figure 5:**
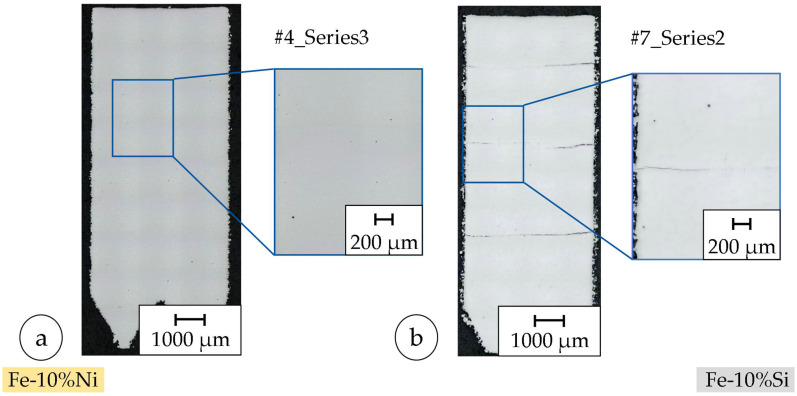
Picture of samples with highest density of Fe-10%N study (**a**) and highest density of Fe-10%Si study (**b**).

Crack-free production of the samples made of Fe-10%Ni was achieved using the parameter set shown in Table 6. Although some specimens exhibited even higher rel. densities, they showed significant cracking and were, therefore, not considered for the fabrication of the toroidal samples. This is essential for the subsequent production of the toroids because cracks have a negative influence on the magnetic properties [82]. However, cracks were found in the sample made from Fe-10%Si specimens, with the highest density showing significant cracking; to fabricate toroids, we, therefore, chose a less crack-prone but slightly lower-density parameter set, which inherently compromises magnetic performance (air-gap effect or higher losses) as shown in Figure 5. A possible solution to this effect is to pre-heat the build chamber and substrate plate, and further adopt in situ scan strategy adaptation to reduce the thermal gradient and therefore the thermal stress during the manufacturing process [47,66,72,83].

A (pre)-heatable building platform was not available in the system used to counteract the fracture/crack formation. Instead, a parameter set (cf. Table 6; Specimen 7, second iteration) with a lower rel. density was selected to produce the toroids (cf. Table 6; Fe-10%Si), as the fracture formation was quantitatively lower.

To improve model accuracy and stability, the logarithm of the rel. density was used as the target variable instead of the rel. density itself. This approach helps to linearize the otherwise nonlinear relationship between process parameters and density, enhances the model’s sensitivity in the high-density range, and ensures numerical stability by better handling small variations near full density.

In Table 7, the results of the chemical analysis performed using X-ray fluorescence analysis are shown. The results are based on spot measurements taken on the fabricated toroids and provide insights into their elemental composition.

#### 3.2.1. Fe-10%Ni Samples

Utilizing the AI-based optimization software, initially, a regression model with a coefficient of determination (CV-R^2^) of 0.55 and a standard deviation (CV-R^2^-std) of 0.25 was developed. The analysis identified scan speed as the most influential parameter affecting logarithmic porosity, followed by hatch distance and laser power, based on their Gini values (0.69, 0.16, and 0.15, respectively) [69]. The Gini importance—also known as mean decrease in impurity—quantifies how much a variable contributes to reducing the prediction error in decision tree-based models. A higher Gini value indicates that a parameter plays a more significant role in splitting the data and improving model accuracy. In this case, scan speed had by far the largest impact on predicting porosity behavior.

Since the target rel. density was not achieved initially, a second iteration was performed using “Advanced Space Filling Initialization” (ASFI). The second iteration prioritized parameter combinations with a laser power of 120 W due to higher average rel. densities observed in the first iteration. The model quality of the combined first and second iterations yielded a CV-R^2^ of 0.5 and a reduced standard deviation of 0.2. Adjusted Gini values indicated that laser power’s influence increased, surpassing hatch distance but still secondary to scan speed (cf. Table 8).

**Figure 6:**
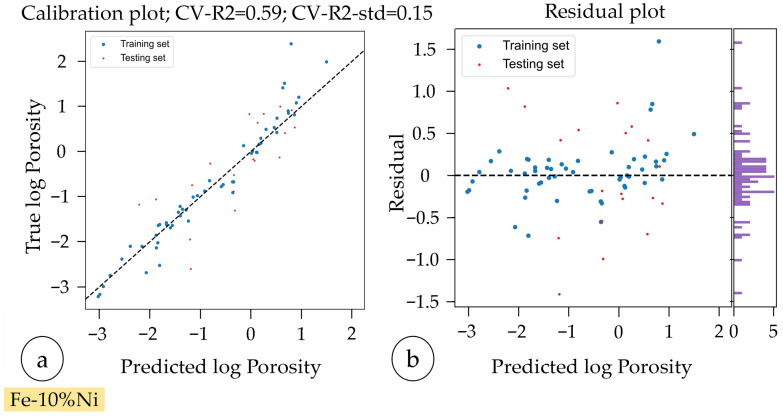
Visualization of the model quality of the parameter study of Fe-10%Ni: (**a**) calibration plot and (**b**) residual plot with residual histogram illustrating error distribution and model bias (purple).

In the third iteration, process optimization was successfully achieved, with the three samples exceeding the target rel. density of ≥99.95% under fixed conditions of 180 W laser power and 60 µm hatch distance. The predictive model developed in this iteration showed improved performance, with a cross-validated coefficient of determination (CV-R^2^) of 0.59 and a reduced standard deviation (CV-R^2^-std) of 0.15, indicating an enhanced balance between model accuracy and robustness. This improvement was supported by the integration of 20 additional measurement points into the training dataset (cf. Figure 6). The observed deviations in the distribution in the purple histogramm indicates systematic bias in the model used.

**Figure 7:**
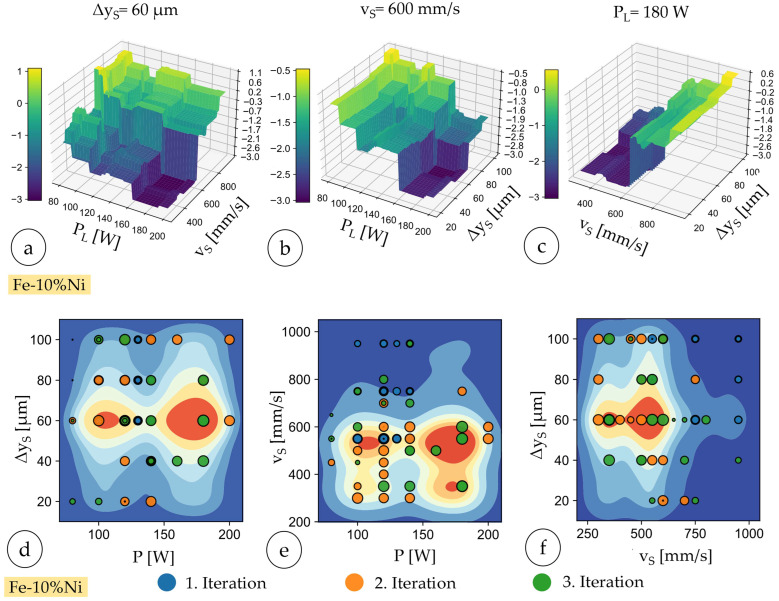
Three-dimensional mesh diagram of the predicted logarithmic porosity of sample no. 4 from the third iteration: (**a**) P_L_xv_s_ and Δy_s_ = const., (**b**) P_L_xΔy_s_ v_s_. = const., (**c**) v_s_xΔy_s_ and P_L_ = const.; (**d**–**f**) heatmaps for the 1, 2, and 3. Iterations within the Fe-10%Ni study.

Final recalculations of Gini values affirmed the primary influence of scan speed (0.58), followed by hatch distance (0.37) and laser power (0.08), indicating consistent parameter importance throughout all the iterations. Three-dimensional modeling highlighted the nonlinear interactions of parameters, effectively visualizing regions with optimal rel. density. Figure 7a–c show the predicted values of logarithmic porosity (a–c) and rel. density (d–f). In waterfall diagrams (a–c), the dark blue regions represent areas of high rel. density, indicating optimal parameter combinations for laser power, scan speed, and hatch distance. In heatmaps (d–f), the dark red regions indicate areas with a high predicted probability of achieving samples with high rel. density based on the underlying regression model and its parameter interaction analysis. One parameter is fixed to create the three-dimensional mesh diagram. The value of the fixed parameter corresponds to the respective value of the parameter combination of sample no. 4 from the third iteration (cf. Table 6 and Table 8).

The heatmaps (cf. Figure 7d–f) showing the predicted rel. density of the Fe-10%Ni specimens as a function of process parameters across three optimization iterations show two-dimensional projections of the parameter space: (d) P_L_ vs. Δy_s_, (e) P_L_ vs. scan speed vs., and (f) scan speed vs. vs. Δy_s_. Data points are color-coded by optimization iteration: dark blue for the first iteration, orange for the second iteration, and green for the third iteration. The progressive clustering of points within the red regions highlights the optimization’s success in converging towards parameter combinations yielding high relative densities.

The parameter study was considered complete after the three samples from the third iteration exceeded the target rel. density threshold of ≥99.95% (cf. Table 9).

#### 3.2.2. Fe-10%Si Samples

An iterative parameter study was also conducted for Fe-10%Si using the same AI-based optimization software. The initial regression model presented a CV-R^2^ of 0.65 and a CV-R^2^-std of 0.19, indicating a robust initial model fit. The analysis showed laser power as the most influential parameter affecting logarithmic porosity, followed by scan speed and hatch distance, with respective Gini values of 0.4 (P_L_), 0.35 (v_s_), and 0.25 (Δy_s_).

As the target rel. density was initially achieved in only one sample, a second iteration was necessary. The second iteration focused on parameter combinations around a laser power of 140 W, scan speed of 550 mm/s, and hatch distance of 60 µm, resulting in two samples surpassing the targeted rel. density of ≥99.95%, thereby concluding the parameter study.

**Figure 8:**
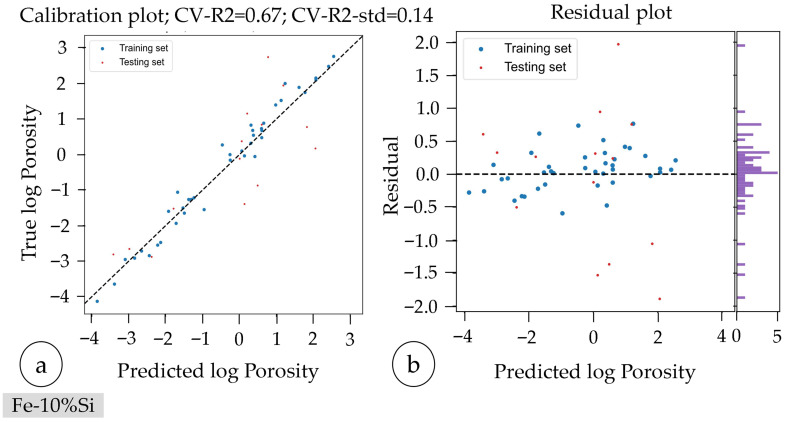
Visualization of the model quality of the parameter study of Fe-10%Si: (**a**) calibration plot and (**b**) residual plot with residual histogram illustrating error distribution and model bias (purple).

The final regression model exhibited slightly improved quality (CV-R^2^ = 0.67, CV-R^2^-std = 0.14), benefiting from the addition of the second iteration’s measurement points (cf. Figure 8). The observed roughly symmetric distribution in the purple histogramm indicates minimal systematic bias and therefore lower potential model deviations compared to the Fe-10%Ni Series.

The final Gini values showed minor changes; laser power increased slightly in importance (0.23), while scan speed (0.44) and hatch distance (0.33) decreased marginally, maintaining their relative order of influence (cf. Table 10).

**Figure 9:**
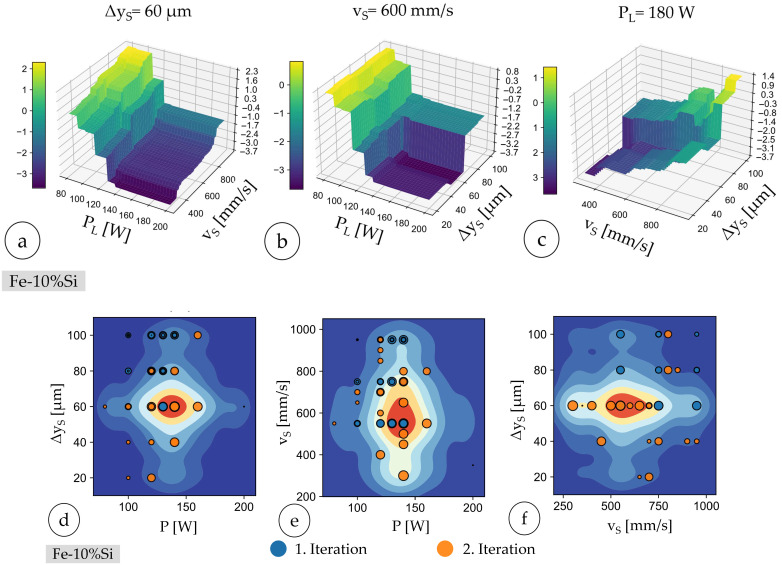
Three-dimensional mesh diagram of the predicted logarithmic porosity of sample no. 2 from the second iteration: (**a**) P_L_xv_s_ and Δy_s_ = const.; (**b**) P_L_xΔy_s_ and v_s_. = const., and (**c**) v_s_xΔy_s_ and v_s_ = const.; heatmaps for the 1., 2., (**d**–**f**) and 3. Iterations within the Fe-10%Si study.

The three-dimensional visualization confirmed the nonlinear relationships between the parameters and rel. density, highlighting optimal parameter regions effectively. Figure 9a–c show the predicted values of logarithmic porosity and relative density. One parameter is fixed to create the three-dimensional mesh diagram. The value of the fixed parameter corresponds to the respective value of the parameter combination of sample no. 7 from the second iteration (ref. Table 6 and Table 11).

Analogous to the parameter study of Fe-10%Ni, the areas that are close to the target value (rel. density is larger then 99.95%) are colored red. The relationship between the setting parameters and the rel. density is not linear when processing Fe-10%Si.

The final parameter combination and measured values are listed in Table A2. The parameter study is completed because the relative density of the three samples from the third iteration is ≥99.95% (see Table 11).

### 3.3. Dynamic Differential Scanning Calorimetry

**Figure 10:**
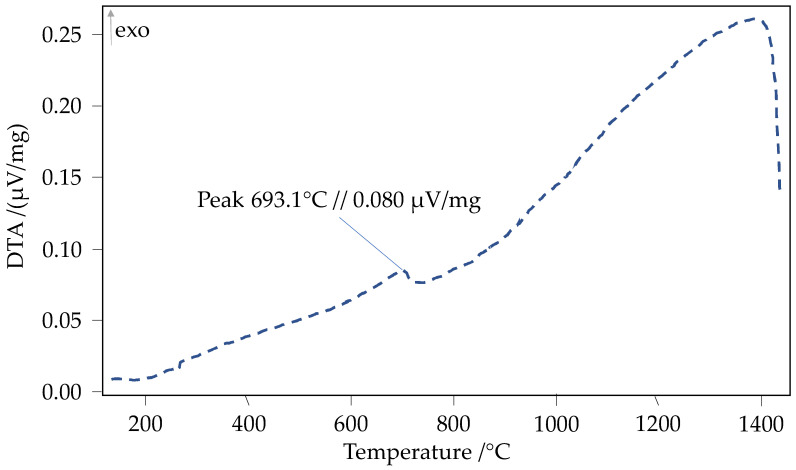
Differential thermal analysis (DTA) of a Fe-10%Si toroid sample: thermal characterization of a material sample over a temperature range from approximately 100 °C to about 1400 °C.

To rule out the possibility that local chemical gradients in the toroidal cores led to partial melting during heat treatment, a differential thermal analysis (DTA) was performed on a sample of the Fe-10%Si ring cores. In the present study, DTA measurements were conducted exclusively on the Fe-10%Si specimens to assess the potential for local Si segregation and partial melting during the applied heat treatment cycle. The resulting thermogram reveals only a subtle exothermic feature at 693.1 °C (0.080 µV/mg) and lacks any pronounced endothermic or melting peaks up to 1400 °C (cf. Figure 10). These findings demonstrate that the Si distribution within the toroidal cores remains chemically homogeneous throughout the thermal protocol. Collectively, the DTA data confirm the absence of significant chemical gradients or partial melting in the Fe-Si cores, thereby validating the integrity of their microstructure after heat treatment. The DTA curve exhibits a continuous upward trend with only a minor and barely discernible peak at 693.1 °C (0.080 µV/mg), and no distinct melting peaks at varying temperatures. This indicates the absence of significant chemical inhomogeneities within the material (cf. Figure 10).

In summary, the graph clearly illustrates a significant thermal transformation at approximately 693.1 °C, and at least one additional pronounced thermal event above approximately 1000 °C, indicating a strong exothermic reaction.

### 3.4. Soft-Magnetic Properties and Heat Treatments

**Figure 11:**
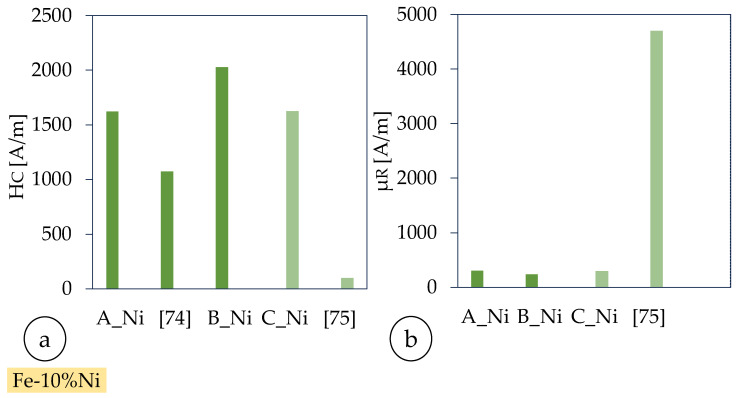

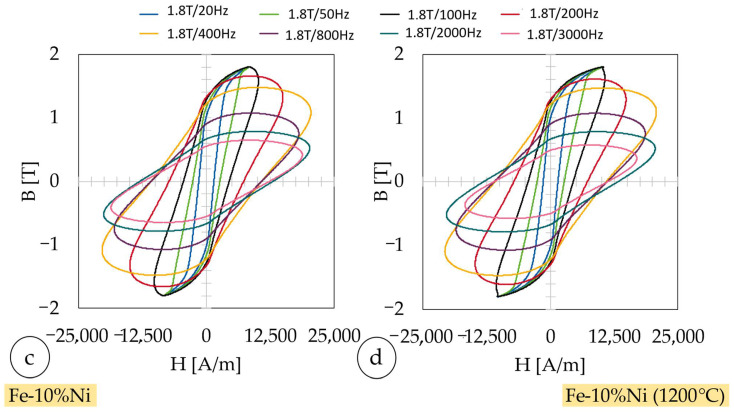
Magnetic properties (hysteresis curves) of additively manufactured Fe-10%Ni toroid at f = 50 Hz and J = 1 T [84,85] as a reference PFB-LB/M manufactured with pre-alloyed (**a**) coercivity (Hc), (**b**) permeability(µR); magnetic field strength (H) over magnetic flux density (B) with demagnetization curves for the Fe-10%Ni-torids (**c**), and heat-treated Fe-10%Ni-torids (**d**).

The coercivity of sample A_Ni_ is approximately 50% greater than the reference value (cf. Figure 11). However, no value is given for the permeability number. The coercivity of the heat-treated Fe-50%Ni alloy is about 6% of the measured value of sample C_Ni_. At the same time, the relative permeability is about 16 times larger than that of sample C_Ni_. Thus, the measured soft-magnetic properties are lower than those of the reference values and, therefore, undesirable. Furthermore, no improvements due to heat treatment are detectable.

Figure 11c,d illustrate the demagnetization curves of Fe-10%Ni toroids, where Figure 11c represents the as-fabricated state and Figure 11d shows the heat-treated condition at 1200 °C. The magnetic response is characterized by the B-H loops obtained under varying excitation frequencies and field strengths. The general shape of the hysteresis loops indicates a soft-magnetic behavior for both conditions, as evidenced by the relatively narrow loops and low coercivity.

A B–H loop (or hysteresis loop) is a plot of the magnetic flux density B versus the applied magnetic field strength H as you cycle the field up and down. H (in A/m) is the intensity of the external magnetic field you apply to the sample, and B (in T or mT) is the resulting magnetic flux density (or magnetization) inside the material.

As you ramp H positively and then negatively, the material’s B does not retrace the same path—this “lag” is what gives the characteristic loop shape. The area inside the loop corresponds to energy loss per cycle, and the loop’s width (coercivity) tells you how “hard” or “soft” the magnetic material is. Narrow loops with small coercive fields are the hallmark of soft magnets.

Notably, the remanent magnetization (magnetic remanence) of the heat-treated Fe-10%Ni samples (cf. Figure 11d) remains nearly unchanged in comparison to the untreated state (Figure 11c). Despite thermal processing, which often alters grain structure and phase composition, the material retains its ability to maintain a high remanent magnetic flux density across all the tested frequencies. This suggests that the primary magnetic domain structure remains stable and is not significantly affected by the annealing treatment at 1200 °C.

Furthermore, both sample conditions exhibit frequency-dependent behavior, with higher frequencies leading to broader loops and reduced peak magnetic flux density. This frequency-induced loss is typical for soft-magnetic materials due to eddy current and hysteresis losses.

In Table 12, the magnetic properties of different literature Fe-Ni alloys are summarized. It should be noted that the Ni content of one of the alloys listed (Fe-50%Ni) is 40% higher than that of the Fe-Ni alloy used, which increases permeability, but also electrical conductivity and thus eddy current losses [86]. Therefore, the values from the literature review are only used as a reference. Overall, our AI-optimized PBF-LB/M processing and subsequent heat treatments yield Fe-10%Ni soft-magnetic properties that compare favorably with—and in certain respects surpass—those reported in conventional and AM literature. Although our as-fabricated coercivity (Hc ≈ 1621 A/m) is higher than the 1074 A/m typically observed for conventionally cast Fe-10%Ni, this elevation can be directly linked to the refined AM microstructure and residual stresses inherent to PBF-LB/M. Crucially, high-temperature annealing (1 h at 1200 °C) restores permeability (µR ≈ 299) to values nearly identical to the as-built state, demonstrating effective stress relief without excessive grain growth. By contrast, Fe-50%Ni alloys achieve much higher permeability (µR ≈ 4700) and lower coercivity (Hc ≈ 100 A/m), but at the expense of increased eddy current losses due to greater electrical conductivity. In this context, our results highlight that the Fe-10%Ni composition, processed under xT-Saam-optimized parameters and judiciously annealed, strikes an optimal balance between magnetic softness, energy loss, and manufacturability, positioning it as a highly competitive candidate for next-generation soft-magnetic components.

**Figure 12:**
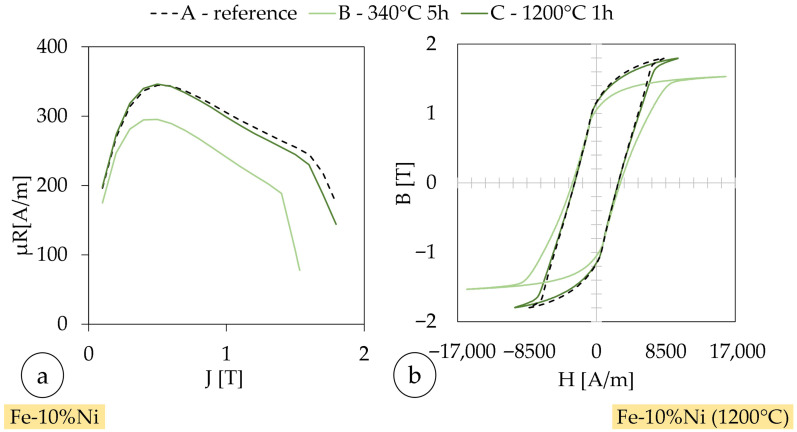
Relative permeability in dependence of polarization of Fe-10%Ni at (**a**) f = 1 Hz 10; samples as-built and (**b**) heat-treated (1200 °C).

Figure 12 presents the magnetic behavior of Fe-10%Ni samples in two complementary ways: (a) shows µ_r_ as a function of J, while (b) depicts the corresponding B-H hysteresis loops. The samples represent different processing states: A_Ni_ is the as-fabricated condition, B_Ni_ was heat-treated at 340 °C for 5 h, and C_Ni_ at 1200 °C for 1 h. (cf. Table 12) In subfigure (a), sample B shows a significantly lower relative permeability across the entire polarization range compared to samples A_Ni_ and C_Ni_. This indicates that the thermal treatment at 340 °C was ineffective in improving the magnetic softness and may have even introduced additional microstructural inhomogeneities (e.g., partial stress relaxation without grain growth). In contrast, sample C_Ni_, which underwent a high-temperature heat treatment, shows a recovery of permeability values comparable to the reference sample A, suggesting partial grain growth and reduced pinning sites for domain wall movement. Figure 11c,d supports this interpretation: the B-H loop for sample B_Ni_ is wider and flatter, indicating increased coercivity and reduced saturation behavior, both of which are signs of higher hysteresis losses. The loops of samples A_Ni_ and C_Ni_ are narrower and more ‘ideal’, suggesting better soft-magnetic performance and lower energy dissipation per magnetization cycle.

From this, it can be concluded that sample B_Ni_ exhibits increased iron losses, likely due to suboptimal microstructural evolution during low-temperature annealing. The combination of high coercivity, reduced permeability, and a broad hysteresis loop points to elevated hysteresis and eddy current losses. Sample C_Ni_, while not showing a dramatic improvement over A_Ni_, benefits from the high-temperature treatment, which partially restores magnetic performance. These findings underscore the importance of properly tailored heat treatments to achieve favorable microstructural conditions, especially grain size enlargement and orientation, for minimizing iron losses in soft-magnetic materials. The low coercivity of samples A_Ni_-C_Ni_ is due to the comparatively high core losses [21]. Core losses refer to the energy dissipated due to hysteresis and eddy current effects. Due to the high coercivity compared to the reference values, the permeability number is negatively affected [21,87,88]. The observed high iron losses in the investigated Fe-based samples can be attributed primarily to the microstructural state of the material. Iron losses, comprising both hysteresis and eddy current losses, are known to be strongly influenced by grain size, crystallographic texture, and electrical resistivity. In the as-fabricated state, the fine-grained microstructure with randomly oriented grains leads to an increased density of domain wall pinning sites, thereby raising the energy required for magnetization reversal and contributing to elevated hysteresis losses. Furthermore, insufficient grain growth and the absence of a pronounced texture after thermal treatment can limit the expected improvement in magnetic softness. PBF-LB/M leaves a melt-pool/cellular substructure, residual porosity, and rough internal surfaces; moreover, minor inclusion populations associated with in situ blending persist after annealing. These features act as efficient domain-wall pinning sites and are not eliminated by a single short high-temperature hold. In parallel, the build-direction columnar grains and solidification-induced texture are only partially recovered; stress is relieved, but crystallographic orientations are not sufficiently randomized, and grains do not coarsen enough to facilitate easy wall motion. As a result, macroscopic coercivity remains governed by persistent pinning and texture rather than by dislocation density alone. Further reduction in Hc will require thermal schedules that disrupt melt-pool substructure and promote recrystallization/texture randomization (e.g., stepwise stress-relief → recrystallization or field annealing), potentially complemented by process-side measures (pre-heating, scan-strategy/porosity mitigation). In addition, the electrical conductivity of the alloy, particularly in Ni-rich compositions, facilitates the formation of eddy currents under alternating magnetic fields, further increasing the total core losses. These combined effects result in a degradation of the magnetic performance, as evidenced by reduced permeability and higher coercivity, and emphasize the critical role of tailored microstructure—especially grain size enlargement and orientation control—in minimizing iron losses in soft-magnetic materials.

The coercivity of the Fe-Si alloy used decreases after the heat treatment (cf. Figure 12). The minimum is HC = 300 A/m for sample C_Si_. This behavior is consistent with the relationship between thermal processing and magnetic softness described in Section 2.8. The relative permeability µ_R_ increases correspondingly, attaining a maximum of µ_R_ = 1114 for sample C_Si_—nearly three times higher than that of the untreated sample. At the same time, a favorable grain orientation with respect to the magnetic field direction minimizes magnetocrystalline anisotropy barriers, further enhancing the permeability. Thus, it is the deliberate control of microstructural features, especially grain size and orientation, that governs the soft-magnetic behavior of the alloy after heat treatment. The observed improvements in magnetic performance in sample C_Si_ are therefore a direct manifestation of targeted microstructural optimization.

**Figure 13:**
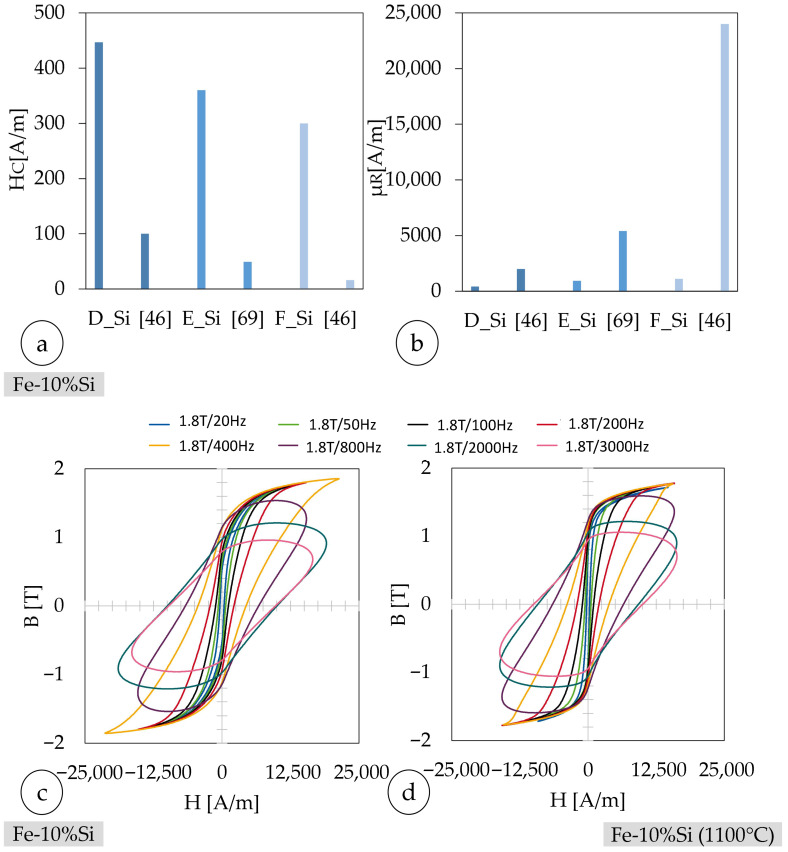
Magnetic properties (hysteresis curves) of Fe-10%Si at f = 50 Hz and J = 1 T [46,75] as a reference PFB-LB/M manufactured with pre-alloyed powder (**a**) coercivity (Hc), (**b**) *permeability(*µ_R_); (**c**) magnetic field strength (H) over magnetic flux density (B) with demagnetization curves for the Fe-10%Si-torids, (**d**) and heat-treated Fe-10%Si-torids.

Figure 13 displays the demagnetization curves of Fe-10%Si toroids, with (c) representing the untreated samples and (d) showing the heat-treated samples after annealing at 1100 °C. The hysteresis loops recorded under various frequencies reveal the magnetic behavior of the material in both states.

The remanent magnetization (B_r_) remains largely stable even after heat treatment, as the comparison between the untreated and annealed samples shows. This indicates that the microstructural changes induced by annealing at 1100 °C do not significantly affect the domain alignment or saturation retention of the Fe-10%Si alloy. The near-identical remanent values across a range of frequencies support the conclusion that the material retains its soft-magnetic properties post-treatment.

As observed with other soft-magnetic materials, the hysteresis loops become progressively wider and less saturated at higher frequencies, primarily due to increased eddy current losses and dynamic domain wall motion resistance. The comparison also shows that while heat treatment may slightly reduce coercivity and energy loss per cycle, it does not adversely affect the material’s ability to retain magnetic flux.

Overall, both the as-fabricated and thermally treated Fe-10%Si samples exhibit good magnetic retention and typical frequency-dependent behavior, making them suitable candidates for applications requiring soft-magnetic performance under dynamic excitation.

**Figure 14:**
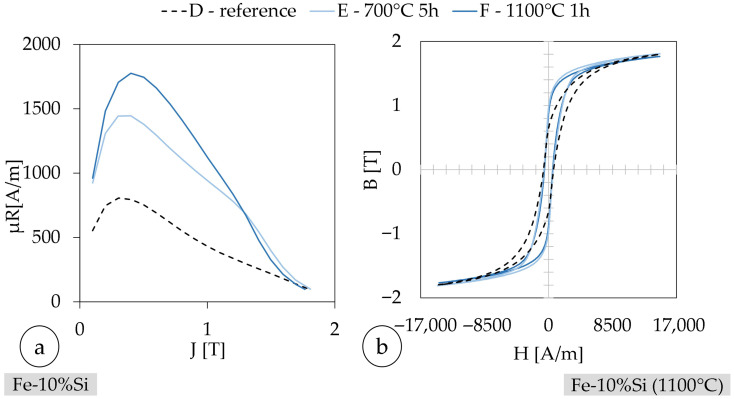
Relative permeability in dependence of polarization of Fe-10%Si at (**a**) f = 1 Hz; samples as-built and (**b**) heat-treated (1100 °C).

Figure 14 shows the magnetic behavior of Fe-10%Si samples in two complementary ways: (a) shows µr as a function of J, while (b) depicts the corresponding B-H hysteresis loops. The samples represent different processing states: A_Si_ is the as-fabricated condition, and C_Si_ at 1100 °C for 1 h. (cf. Table 13).

In Table 13, the magnetic properties of a similar Fe-Si alloy from the literature with a Si content of 6.9%wt ≅ 13.5%at are summarized. It should be noted that as the Si content increases, the coercivity decreases and the permeability increases [82]. For this reason, the values are only used as a reference.

The coercivity of sample A_Si_ is larger than the reference value (ref. Table 13). The relative permeability of the reference is about five times larger compared to that of sample A_Si_ [54,82]. The measured coercivity of Fe-13.5%Si after heat treatment at 700 °C for 5 h was about half the measured value of untreated Fe-13.5Si. In the present study, the same heat treatment also led to a decrease in coercivity, but not to the same extent. Compared to the untreated reference alloy with 13.5% Si, which has a coercivity of 100 A/m, this is still about three times higher. Thus, while the thermal treatment in this work led to a considerable improvement, particularly by reducing coercivity by nearly one order of magnitude, the absolute values reported in the literature are not fully reached. This discrepancy can be attributed to differences in chemical composition (higher Si content in the literature alloys), potential inhomogeneities from powder blending, and the formation of cracks during processing, which likely reduce the effectiveness of the heat treatment. The benchmarks cited use up to Fe-50%Ni or Fe-13.5%Si; both compositions intrinsically enable lower coercivity/higher permeability than the used Fe-10%Ni and Fe-10%Si, even after annealing. The permeability is approximately doubled after heat treatment, but the absolute value achieved was only about one-fifth of the reference value for Fe-13.5%Si. These proportions are also present after heat treatment at 1150 °C for 1 h. The coercivity of Fe-13.5%Si was only about one-third of the measured value after heat treatment at 700 °C for 5 h. The permeability of Fe-13.5%Si is almost 24 times larger than that of sample C_Si_ after heat treatment at 1150 °C for 1 h. Overall, the measured magnetic properties are lower than the reference values. In addition, the samples cannot be produced crack-free with the determined parameter combination (cf. Figure 5). Cracks act as local air gaps with low magnetic permeability, significantly disturbing the magnetic flux path. As a result, additional magnetic energy is required to overcome these discontinuities, leading to increased coercivity and higher hysteresis losses [54,82]. As a result, the coercivity increases and the permeability number decreases [54,82,89].

The physical and magnetic properties of the materials correlate with each other [32,82]. Since soft magnets are characterized by low coercivity and high permeability compared to other magnets, the magnetic properties are improved by a reduction in internal stresses and grain growth. Furthermore, core losses are reduced. These relate to the total energy lost through the generation of heat. It is the loss that occurs in a magnetic core due to alternating magnetization, which is the sum of the hysteresis loss and the eddy current loss. One reason for this is that the expansion of the domains is hindered by internal stresses and grain boundaries. This can be compensated for by heat treatment of the geometry [45,46,75,76,82]. A plausible reason for the low magnetic properties is the orientation of the toroids during the PBF-LB/M process. In the present work, the bases of the samples are located in the XY-plane, and the specimens are built along the Z-direction using the PBF-LB/M process. Due to the high cooling rates and steep thermal gradients inherent to the process, a fine-grained microstructure typically forms within the XY-plane. In contrast, elongated grains or directional grain growth may develop along the build direction (*Z*-axis), which corresponds to the YZ- and XZ-planes. This phenomenon is characteristic of the epitaxial solidification behavior observed in additively manufactured materials, where the grain structure reflects the thermal history of the process.

It is important to note that post-processing heat treatments and stress-relief annealing (ref. Table 13) can significantly influence the as-built microstructure. Depending on the applied parameters, such treatments may lead to partial or complete recrystallization and thus reduce the anisotropy of the grain structure. However, in many cases, residual texture and morphological differences between planes may still remain, which can impact the mechanical properties of the material [90,91,92,93,94].

As a result, the orientation of the magnetic flux density during the magnetization process of the toroids is perpendicular to the XZ-plane. Due to the fine-grained structure present there, the propagation of the domains is impeded, and thus the coercivity increases and the permeability number decreases. However, the comparatively coarse-grained structure of the YZ-plane can be utilized by rotating the toroids so that the base lies in this plane. This should decrease the coercivity and increase the permeability number [77,82].

## 4. Conclusions and Outlook

The objective of the present work was to develop an approach for processing soft-magnetic materials using PBF-LB/M. Fe-10%Ni and Fe-10%Si were used as materials. First, a parameter study was performed to develop suitable processing parameters with the rel. density as the target parameter because it correlates with the magnetic properties. The results of the parameter study, namely, the identified processing parameters for the materials used, which are P_L_ = 180 W, vs. = 500 mm/s, and Δy_S_ = 40 µm for Fe-10%Ni and, respectively, P_L_ = 140 W, vs. = 300 mm/s, and Δy_S_ = 60 µm for Fe-10%Si, enabled the production of samples with promising structural integrity.

Subsequently, toroids were fabricated using the developed setting parameters and heat-treated to improve the magnetic properties, and measured in this regard. The magnetic characteristics of Fe-10%Ni were HC = 1621 A/m and µR = 305, and HC = 300 A/m and µR = 1114 of Fe-10%Si. The magnetic properties obtained here are lower than literature values, as expected for powder-blend specimens compared to pre-alloyed powders. These results provide baseline data, defining the processing limits for in situ alloying and highlighting the need for strategies such as pre-heating, heat treatments, or tailored feedstocks to approach pre-alloyed performance. For Fe-10%Ni, heat treatment showed no improvement, unlike Fe-10%Si, with grain growth having a stronger negative effect than stress relief. Using in situ powder blends and AI-guided parameter development, a quantitative processing window was established for Fe-10 at% Ni and Fe-10 at% Si in PBF-LB/M, including heat/mesh-map evidence for parameter sensitivities toward ≥ 99.95% relative density. Crack susceptibility in Fe-10% Si enforced a practical trade-off in terms of fabrication. Across the batches the PSD shifted to coarser fractions due to agglomeration and fine-fraction removal during processing, informing powder handling and parameter transfer. Magnetically, Fe-10% Ni achieved ~99.96% rel. density yet showed A H_c_ ≈ 1621 A/m and *μ*_R_ ≈ 305 A/m with only limited change after 1200 °C/1 h, while Fe-10% Si reached *H*_c_ ≈ 300 A/m and μ_R_ ≈ 1114 after 1100 °C/1 h; these results delineate the current performance ceiling for blend-based PBF-LB/M without pre-heating and rationalize gaps to the Fe-13.5% Si literature via composition and crack effects. Microstructure, texture, and build orientation are affected by the build direction. The toroids were built on the XY-plane and grown along Z; AM-typical epitaxial grain growth and residual texture can hinder domain-wall motion until sufficiently recrystallized. The observed limited improvement after annealing (Fe-Ni: 1 h @ 1200 °C; Fe-Si: 1 h @ 1100 °C) indicates insufficient grain coarsening/texture optimization for maximum softness. The expected improvement in magnetic properties, particularly relative permeability, was not achieved. The powder blends likely introduced local chemical inhomogeneities, suggesting that pre-alloyed powders should be explored to enhance homogeneity. For Fe-10%Ni, the applied heat treatment was insufficient, and systematic studies (e.g., using Xt-Saam) are needed to optimize process conditions. Although Fe-10%Si responded positively to heat treatment, its performance was limited by sample cracking. Pre-heating and pre-alloyed powders remain promising strategies to reduce thermal gradients, improve microstructural uniformity, and enhance magnetic properties [95,96,97]. These techniques were not included in our current parameter study. Future work will incorporate powder bed pre-heating and pre-alloyed feedstocks to evaluate their synergistic effects on densification, microstructure, and soft-magnetic properties [82,86]. With the implementation of these suggestions, it should be possible to produce soft magnets by means of PBF-LB/M in further iterations, with equivalent properties to conventionally manufactured ones. Despite the challenges identified, this study demonstrates the fundamental feasibility of producing soft-magnetic components via PBF-LB/M. With targeted improvements, such as optimized heat treatment protocols, improved chemical homogeneity, and defect prevention through pre-heating, it is expected that magnetic properties equivalent to those of conventionally manufactured materials can be achieved in future work.

While previous works have demonstrated that pre-alloyed powders and powder bed pre-heating yield superior magnetic properties in PBF-LB/M of soft-magnetic alloys, the potential of in situ alloying via powder blends remains less explored. In particular, no study to date has applied AI-driven parameter optimization to systematically map the processing limits of Fe-10 at% Ni and Fe-10 at% Si blends without pre-heating or pre-alloying. By doing so, we aim to (i) establish quantitative benchmarks for achievable density and magnetic performance, (ii) identify process–structure–property relationships specific to powder blends, and (iii) provide guidance on when powder-blend strategies can be a cost-effective alternative or require supplementation with pre-alloyed feedstocks.

In this work, Fe-10 at% Ni reached a maximum relative density of 99.96%, yet the as-built toroid exhibited Hc ≈ 1621 A/m and µR ≈ 305 with only limited improvement after 1200 °C/1 h, whereas Fe-10 at% Si improved to Hc ≈ 300 A/m and µR ≈ 1114 after 1100 °C/1 h but still trailed Fe-13.5% Si benchmarks—consistent with lower Si content and processing-induced cracking. Taken together, these data quantify the current ceiling of blend-based PBF-LB/M without pre-heating and motivate targeted improvements:

(i) Pre-heating and/or stress-relief plus recrystallization anneals tailored to each alloy,
(ii) Crack-mitigation for Fe-Si (e.g., scan strategy, platform heating), and
(iii) Optional use of pre-alloyed feedstocks where maximum softness is mandatory.

## Figures and Tables

**Table 1:** Process window for determining the setting parameters.

/	$P_{L}\left[W\right]$	$v_{S}\left[\frac{mm}{s}\right]$	$\Delta y_{S}\left[µm\right]$	$D_{S}[µm]$
Process window	80–200	250–1000	20–100	30
Step size	20	50	20	-

**Table 2:** Magnetic frequencies and polarization ranges used in the Fe-10%Ni and Fe-10%Si study.

Magnetization Frequencies	Polarization Range
20 Hz	0.1–1.8 T
400 Hz	0.1–1.8 T
800 Hz	0.1–1.8 T
2000 Hz	0.1–1.5 T
3000 Hz	0.1–1.0 T

**Table 3:** Independent heat treatments used in study for the Fe-10%Ni and Fe-10%Si toroids.

Chem. Compositon	Toroid/Specimen	Designation
Fe-10%Ni	untreated	A_Ni_
	340 °C for 5 h	B_Ni_
	1200 °C for 1 h	C_Ni_
Fe-10%Si	untreated	As_i_
	700 °C for 5 h	B_Si_
	1100 °C for 1 h	C_Si_

**Table 4:** Powder specifications from manufacturers.

Element	Manufacturer	PSD
Fe	TLS Technik GmbH & Co. Spezialpulver KG (Bitterfeld, Germany)	<45 µm
Ni	thyssenkrupp Materials Schweiz AG (Bad Oeynhausen, Germany)	10–45 µm
Si	TLS Technik GmbH & Co. Spezialpulver KG	10–45 µm

**Table 5:** Particle size distribution (PSD) values of the blended powders used.

Chem. Composition	Batch	D_10_ [µm]	D_50_ [µm]	D_90_ [µm]
Fe-10%Ni (at%)	A	12.34	29.42	47.92
	B	11.77	28.45	50.71
Fe-10%Si (at%)	A	11.68	27.10	46.82
	B	14.06	31.76	52.86

**Table 6:** Samples with the highest rel. density of both procedures used for a parameter study FeNi and FeSi.

Regression	Specimen Number	Iteration	$P_{L}\left[W\right]$	$v_{S}\left[\frac{mm}{s}\right]$	$\Delta y_{S}\left[µm\right]$	Rel. Density [%]
Fe-10%Ni	4	3	180	600	60	99.960
Fe-10%Si	28	1	140	550	60	99.974

**Table 7:** Complete overview of the chemical composition of the Fe-10%Ni and Fe-10%Si toroids.

Element/Share	Fe-10%Ni [wt%]	Fe-10%Si [wt%]
Ni	10.144	0.099
Si	0.089	11.884
Fe	86.893	86.792
Cr	0.012	0.014
Mo	0.018	0.016
Al	0.829	0.840
Ti	0.211	0.239
Co	0.03	0.03
Cu	0.094	0.099
W	0.090	0.086

**Table 8:** Gini coefficients of the first and second iterations from Fe-10%Ni.

Parameter	Gini Coefficient
$v_{S}$	0.62
$\Delta y_{S}$	0.28
$P_{L}$	0.1

**Table 9:** Parameter combinations of xT Saam of Fe-10%Ni with relative density ≥ 99.95%.

Specimen #	Iteration	$P_{L}\left[W\right]$	$v_{S}\left[\frac{mm}{s}\right]$	$\Delta y_{S}\left[µm\right]$	Rel. Density [%]
4	3	180	600	60	99.960
18	3	180	550	60	99.958
6	3	180	350	60	99.950

**Table 10:** Gini coefficients of the first and second iterations from Fe-10%Si.

Parameter	Gini Coefficient
$v_{S}$	0.44
$\Delta y_{S}$	0.33
$P_{L}$	0.23

**Table 11:** Parameter combinations of xT Saam of Fe-10%Si with rel. density ≥ 99.95%.

Specimen #	Iteration	$P_{L}\left[W\right]$	$v_{S}\left[\frac{mm}{s}\right]$	$\Delta y_{S}\left[µm\right]$	Rel. Density [%]
7	2	140	300	60	99,984
28	1	140	550	60	99,974

**Table 12:** Comparison of soft-magnetic properties: present study vs. literature.

Material	HT	H_c_ [A/m]	µ	Reference
Fe-10%Ni	-	1074	n.a.	[85]
Fe-10%Ni	-	1621	305	A_Ni_
Fe-10%Ni	5 h @ 340 °C	2028	241	B_Ni_
Fe-50%Ni	9 h @ 1200 °C	100	4700	[84]
Fe-10%Ni	1 h @ 1200 °C	1625	299	C_Ni_

**Table 13:** Measured soft-magnetic properties and reference values of Fe-Si alloys.

Material	HT	H_c_ [A/m]	µ	Reference
Fe-13.5%Si	-	100	2000	[75]
Fe-10%Si	-	4471	432	A_Si_
Fe-13.5%Si	5 h @ 700 °C	49–54	4900–5400	[46]
Fe-10%Si	5 h @ 700 °C	360	933	B_Si_
Fe-13.5%Si	1 h @ 1150 °C	16	24000	[75]
Fe-10%Si	1 h @ 1100 °C	300	1114	C_Si_

## Data Availability

The original contributions presented in this study are included in the article. Further inquiries can be directed to the corresponding author.

## Abbreviations

The following abbreviations are used in this manuscript:

Abbreviation	Unit	
B	T	Magnetic flux density
$B_{0}$	T	Magnetic flux density in vacuum
$B_{R}$	T	Remanence flux density
CV		Cross-validation
$D_{S}$	µm	Layer thickness
$d_{S}$	µm	Laser beam diameter
$d_{W}$	µm	Bloch wall width
$E_{M}$	J	Energy of the hysteresis curve
$E_{V}$	J	Volume energy density
f	Hz	Frequency
Fe		Iron
H	$\frac{A}{m}$	Magnetic field strength
$H_{C}$	$\frac{A}{m}$	Coercive field strength
J	T	Polarization
PBF-LB/M		Laser powder bed fusion laser beam/metals
M	$\frac{A}{m}$	Magnetization
$V_{Ar}$	$\frac{L}{min}$	Protective gas volume flow
µ	$\frac{Vs}{Am}$	Permeability
${µ}_{0}$	$\frac{Vs}{Am}$	Magnetic field constant
${µ}_{R}$		Permeability number
Ni		Nickel
$P_{L}$	W	Laser power
$P_{S}$	$\frac{W}{kg}$	Specific iron losses
PSD		Particle size distribution
Rel.		relative
Si		Silicon
T	°C	Temperature
$v_{S}$	$\frac{mm}{s}$	Scanning speed
$\Delta y_{S}$	µm	Hatch distance

## Appendix A

The physical and magnetic properties of the materials correlate with each other (cf. Table A1). Compared to other magnets, soft magnets are characterized by a low coercive field strength and high permeability. These magnetic properties are improved by a reduction in internal stresses and grain growth. Furthermore, iron losses are reduced. One reason for this is that the expansion of the domains is hindered by internal stresses and grain boundaries. This can be compensated for by heat treatment of the geometry under investigation.

**Table A1:** Effect of the physical and chemical characteristics and setting parameters on the magnetic properties (ref. [32,82,98]).

Physical Characteristics	$P_{S}$	$H_{C}$	${µ}_{R}$
↑ Grain size	↓	↓	↑
↑ Internal stresses	↑	↑	↓
↑ Porosity	-	-	↓
↑ cracks	↑	↑	↓
↑ Si-content	↓	↓	↑
↑ Ni-content	↑	↓↑	↑
**Process parameter**			
↑ Pre-heating	↓	↓	↑
↑ Heat treatment	↓	↓	↑

## Appendix B

The table provides an overview of the process parameters associated with various soft-magnetic alloys. It highlights key variables such as laser power, scan speed, layer height, and hatch distance, as reported in the literature or experimental studies.

**Table A2:** Parameter sets of Fe-Ni and Fe-Si alloys from the literature.

Material	Fe 49.5%, Ni 49.4%, Cu 0.4%, Mn 0.3%, Si 0.4%	Fe-30%Ni	Fe-6.9%wt Si	Fe-6.5%wt Si
$P_{L}\left[W\right]$	195	50–100	70	130
$v_{S}\left[\frac{mm}{s}\right]$	800	100–1600	500	1100
$\Delta y_{S}\;\left[µm\right]$	100		60	49
$D_{S}\left[µm\right]$	40	50	25	30
Source	[84]	[99]	[75]	[86]

## References

[1] Fredriksson C. (2019). Sustainability of metal powder additive manufacturing. Procedia Manuf..

[2] Kreinin H., Aigner E. (2022). From “Decent work and economic growth” to “Sustainable work and economic degrowth”: A new framework for SDG 8. Empirica.

[3] Steffen W., Broadgate W., Deutsch L., Gaffney O., Ludwig C. (2015). The trajectory of the Anthropocene: The Great Acceleration. Anthr. Rev..

[4] Hagens N.J. (2020). Economics for the future—Beyond the superorganism. Ecol. Econ..

[5] Intergovernmental Panel on Climate Change (2022). Summary for Policymakers. Global Warming of 1.5 °C: IPCC Special Report on Impacts of Global Warming of 1.5 °C Above Pre-Industrial Levels in Context of Strengthening Response to Climate Change, Sustainable Development, and Efforts to Eradicate Poverty.

[6] Intergovernmental Panel on Climate Change (2014). Climate Change 2013—The Physical Science Basis.

[7] Europäisches Parlament Reduktion von CO_2_-Emissionen: Ziele und Maßnahmen der EU. https://www.europarl.europa.eu/pdfs/news/expert/2018/3/story/20180305STO99003/20180305STO99003_de.pdf.

[8] Intergovernmental Panel on Climate Change (2022). Global Warming of 1.5 °C: IPCC Special Report on Impacts of Global Warming of 1.5 °C Above Pre-Industrial Levels in Context of Strengthening Response to Climate Change, Sustainable Development, and Efforts to Eradicate Poverty.

[9] Kaltschmitt M., Schebek L. (2015). Umweltbewertung für Ingenieure.

[10] Smil V. (2017). Energy and Civilization: A History.

[11] Metal AM Magazine Elkem Develops Iron Silicon Soft Magnetic Powder for Additive Manufacturing of Electrical Motor Components. https://www.metal-am.com/elkem-develops-iron-silicon-soft-magnetic-powder-for-additive-Manufacturing-of-electrical-motor-components/.

[12] Lamichhane T.N., Sethuraman L., Dalagan A., Wang H., Keller J., Paranthaman M.P. (2020). Additive manufacturing of soft magnets for electrical machines—A review. Mater. Today Phys..

[13] Garibaldi M., Gerada C., Ashcroft I., Hague R., Morvan H. The Impact of Additive Manufacturing on the Development of Electrical Machines for MEA Applications: A Feasibility Study. Proceedings of the MEA2015 More Electric Aircraft.

[14] Gargalis L., Madonna V., Giangrande P., Rocca R., Hardy M., Ashcroft I., Galea M., Hague R. (2020). Additive Manufacturing and Testing of a Soft Magnetic Rotor for a Switched Reluctance Motor. IEEE Access.

[15] Pakkanen J. (2018). Designing for Additive Manufacturing—Product and Process Driven Design for Metals and Polymers. Ph.D. Thesis.

[16] Lammers S., Adam G., Schmid H.J., Mrozek R., Oberacker R., Hoffmann M.J. Additive Manufacturing of a lightweight rotor for a permanent magnet synchronous machine. Proceedings of the 2016 6th International Electric Drives Production Conference (EDPC).

[17] Top N., Sahin I., Mangla S.K., Sezer M.D., Kazancoglu Y. (2023). Towards sustainable production for transition to additive manufacturing: A case study in the manufacturing industry. Int. J. Prod. Res..

[18] Fremaux A. (2018). Towards a Critical Theory of the Anthropocene and a Life-affirming Politics A Post-Anthropocentric, Post-Growth, Post-(neo)Liberal Green Republican Analysis. Unpublished Ph.D. Thesis.

[19] VDI ZRE (2024). Brief Analysis No. 35: Resource Efficiency Through Additive Manufacturing.

[20] Gebhardt A., Kessler J., Schwarz A. (2019). Produktgestaltung für die Additive Fertigung.

[21] Pham T., Kwon P., Foster S. (2021). Additive Manufacturing and Topology Optimization of Magnetic Materials for Electrical Machines—A Review. Energies.

[22] Jülich P., Jülich F. Ex-Ante Evaluation für ein Technologietransfer-Programm Leichtbau: Kurzfassung. https://www.bmwk.de/Redaktion/DE/Publikationen/Studien/ex-ante-evaluation-technologietransfer-programm-leichtbau.pdf?__blob=publicationFile&v=6.

[23] Pyrhönen J., Jokinen T., Hrabovcová V. (2008). Design of Rotating Electrical Machines.

[24] Zenou M., Grainger L., Zhang J., Jung Y.-G. (2018). Additive manufacturing of metallic materials. Additive Manufacturing.

[25] Jauer L. (2018). Laser Powder Bed Fusion von Magnesiumlegierungen.

[26] DIN Deutsches Institut für Normung e. V (2022). Additive Fertigung—Grundlagen—Terminologie.

[27] Gebhardt A. (2016). Additive Fertigungsverfahren: Additive Manufacturing und 3D-Drucken für Prototyping—Tooling—Produktion.

[28] Zhang J., Jung Y.-G. (2018). Additive Manufacturing.

[29] van Bracht R. (2019). Das Potenzial der additiven Fertigung: Digitale Technologien im Unternehmenskontext.

[30] Gibson I., Rosen D., Stucker B., Khorasani M. (2021). Additive Manufacturing Technologies.

[31] Joshi S., Martukanitz R.P., Nassar A.R., Michaleris P. (2023). Additive Manufacturing with Metals.

[32] Krings A., Boglietti A., Cavagnino A., Sprague S. (2017). Soft Magnetic Material Status and Trends in Electric Machines. IEEE Trans. Ind. Electron..

[33] VDI (2014). Additive Fertigungsverfahren—Grundlagen, Begriffe, Verfahrensbeschreibung.

[34] Yap C.Y., Chua C.K., Dong Z.L., Liu Z.H., Zhang D.Q., Loh L.E., Sing S.L. (2015). Review of selective laser melting: Materials and applications. Appl. Phys. Rev..

[35] Hagedorn W., Gramlich A., Greiff K., Krupp U. (2022). Alloy and process design of forging steels for better environmental performance. Sustain. Mater. Technol..

[36] Böckin D., Tillman A.-M. (2019). Environmental assessment of additive manufacturing in the automotive industry. J. Clean. Prod..

[37] Schneider G., Goll D., Bernthaler T., Kopp A., Rieger T., Schubert T., Schuller D. (2018). Pulvertechnisch hergestellte Werkstoffe für die Elektromobilität—Teil 2: Magnete. Keram. Z..

[38] Shishkovsky I., Saphronov V. (2016). Peculiarities of selective laser melting process for permalloy powder. Mater. Lett..

[39] Weiss C., Boedger C., Schiefer E., Heussen D., Haefner C.L. Evaluation of the Ecological Footprint for Parts from AlSi10Mg manufactured by Laser Powder Bed Fusion. Proceedings of the 2022 International Solid Freeform Fabrication Symposium.

[40] Wurst J., Mozgova I., Lachmayer R. (2022). Sustainability Assessment of Products manufactured by the Laser Powder Bed Fusion (LPBF) Process. Procedia CIRP.

[41] Ewald S., Kies F., Hermsen S., Voshage M., Haase C., Schleifenbaum J.H. (2019). Rapid Alloy Development of Extremely High-Alloyed Metals Using Powder Blends in Laser Powder Bed Fusion. Materials.

[42] Garrard R., Lynch D., Carter L.N., Adkins N.J., Gie R., Chouteau E., Pambaguian L., Attallah M.M. (2022). Comparison of LPBF processing of AlSi40 alloy using blended and pre-alloyed powder. Addit. Manuf. Lett..

[43] Chen C.-W. (1977). Magnetism and Metallurgy of Soft Magnetic Materials.

[44] Hubert A., Schäfer R. (1998). Magnetic Domains: The Analysis of Magnetic Microstructures.

[45] Adler E., Pfeiffer H. (1974). The influence of grain size and impurities on the magnetic properties of the soft magnetic alloy 47.5% NiFe. IEEE Trans. Magn..

[46] Garibaldi M., Ashcroft I., Hillier N., Harmon S., Hague R. (2018). Relationship between laser energy input, microstructures and magnetic properties of selective laser melted Fe-6.9%wt Si soft magnets. Mater. Charact..

[47] Pant P., Salvemini F., Proper S., Luzin V., Simonsson K., Sjöström S., Hosseini S., Peng R.L., Moverare J. (2022). A study of the influence of novel scan strategies on residual stress and microstructure of L-shaped LPBF IN718 samples. Mater. Des..

[48] Pham M.-S., Dovgyy B., Hooper P.A., Gourlay C.M., Piglione A. (2020). The role of side-branching in microstructure development in laser powder-bed fusion. Nat. Commun..

[49] Sun S.-H., Ishimoto T., Hagihara K., Tsutsumi Y., Hanawa T., Nakano T. (2019). Excellent mechanical and corrosion properties of austenitic stainless steel with a unique crystallographic lamellar microstructure via selective laser melting. Scr. Mater..

[50] Ding J., Shi Y., Chen L., Deng C., Fuh S., Li Y. (2002). A structural, magnetic and microwave study on mechanically milled Fe-based alloy powders. J. Magn. Magn. Mater..

[51] Shokrollahi H. (2009). The magnetic and structural properties of the most important alloys of iron produced by mechanical alloying. Mater. Des..

[52] Pepperhoff W., Acet M. (2000). Konstitution und Magnetismus des Eisens und seiner Legierungen.

[53] Shen X., Sheng H., He Y., Liogas K.A., Boon Lau K., Wang P., Meng F., Chen K., Jia N., Ramamurty U. (2023). Evaluation of microstructure, mechanical and magnetic properties of laser powder bed fused Fe-Si alloy for 3D magnetic flux motor application. Mater. Des..

[54] Goll D., Schuller D., Martinek G., Kunert T., Schurr J., Sinz C., Schubert T., Bernthaler T., Riegel H., Schneider G. (2019). Additive manufacturing of soft magnetic materials and components. Addit. Manuf..

[55] Urban N., Bauch L., Armbruster R., Franke J. Evaluation of Soft Magnetic Ferrosilicon FeSi 6.5 for Laser Beam Melting. Proceedings of the 2019 IEEE 9th International Electric Drives Production Conference (EDPC).

[56] Liogas K.A., Lau K.B., Wang Z., Brown D.N., Polatidis E., Wang P., Korsunsky A.M. (2023). Effect of heat treatment on the microstructure and magnetic properties of laser powder bed fusion processed equiatomic Co-Fe. Addit. Manuf..

[57] Huber F., Rasch M., Schmidt M. (2021). Laser Powder Bed Fusion (PBF-LB/M) Process Strategies for In-Situ Alloy Formation with High-Melting Elements. Metals.

[58] Ali M.H., Sabyrov N., Shehab E. (2022). Powder bed fusion–laser melting (PBF–LM) process: Latest review of materials, process parameter optimization, application, and up-to-date innovative technologies. Prog. Addit. Manuf..

[59] Ciccone F., Bacciaglia A., Ceruti A. (2023). Optimization with artificial intelligence in additive manufacturing: A systematic review. J Braz. Soc. Mech. Sci. Eng..

[60] Dharmadhikari S., Menon N., Basak A. (2023). A reinforcement learning approach for process parameter optimization in additive manufacturing. Addit. Manuf..

[61] Cao Y., Chen C., Xu S., Zhao R., Guo K., Hu T., Liao H., Wang J., Ren Z. (2024). Machine learning assisted prediction and optimization of mechanical properties for laser powder bed fusion of Ti6Al4V alloy. Addit. Manuf..

[62] Tiismus H., Kallaste A., Vaimann T., Lind L., Virro I., Rassõlkin A., Dedova T. (2022). Laser Additively Manufactured Magnetic Core Design and Process for Electrical Machine Applications. Energies.

[63] Babuska T.F., Wilson M.A., Johnson K.L., Whetten S.R., Curry J.F., Rodelas J.M., Atkinson C., Lu P., Chandross M., Krick B.A. (2019). Achieving high strength and ductility in traditionally brittle soft magnetic intermetallics via additive manufacturing. Acta Mater..

[64] Leicht A., Yu C.H., Luzin V., Klement U., Hryha E. (2020). Effect of scan rotation on the microstructure development and mechanical properties of 316L parts produced by laser powder bed fusion. Mater. Charact..

[65] Dixit S., Liu S., Smith P.M., Pradeep S.A. (2024). Effects of scan rotation angle and build orientation on mechanical anisotropy in additive manufacturing 316L stainless steel. J. Manuf. Process..

[66] Jhabvala J., Boillat E., Antignac T., Glardon R. (2010). On the effect of scanning strategies in the selective laser melting process. Virtual Phys. Prototyp..

[67] Wirth F., Frauchiger A., Gutknecht K., Cloots M., Meboldt M., Klahn C. (2021). Influence of the Inert Gas Flow on the Laser Powder Bed Fusion (LPBF) Process. Industrializing Additive Manufacturing.

[68] Shipley H., McDonnell D., Culleton M., Coull R., Lupoi R., O’Donnell G., Trimble D. (2018). Optimisation of process parameters to address fundamental challenges during selective laser melting of Ti-6Al-4V: A review. Int. J. Mach. Tools Manuf..

[69] Shao W., He B., Qiu C., Li Z. (2022). Effect of hatch spacing and laser remelting on the formation of unique crystallographic texture of IN718 superalloy fabricated via laser powder bed fusion. Opt. Laser Technol..

[70] Exponential Technologies Ltd Case Study—93% Reduction of R&D Time in Metal Additive Manufacturing. https://www.x-t.ai/reduction-of-rd-time-in-metal-additive-manufacturing/.

[71] Arısoy Y.M., Criales L.E., Özel T., Lane B., Moylan S., Donmez A. (2017). Influence of scan strategy and process parameters on microstructure and its optimization in additively manufactured nickel alloy 625 via laser powder bed fusion. Int. J. Adv. Manuf. Technol..

[72] Chen C., Xiao Z., Wang Y., Yang X., Zhu H. (2021). Prediction study on in-situ reduction of thermal stress using combined laser beams in laser powder bed fusion. Addit. Manuf..

[73] Paraschiv A., Matache G., Constantin N., Vladut M. (2022). Investigation of Scanning Strategies and Laser Remelting Effects on Top Surface Deformation of Additively Manufactured IN 625. Materials.

[74] Paraschiv A., Matache G., Condruz M.R., Frigioescu T.F., Pambaguian L. (2022). Laser Powder Bed Fusion Process Parameters’ Optimization for Fabrication of Dense IN 625. Materials.

[75] Garibaldi M., Ashcroft I., Lemke J.N., Simonelli M., Hague R. (2018). Effect of annealing on the microstructure and magnetic properties of soft magnetic Fe-Si produced via laser additive manufacturing. Scr. Mater..

[76] Lemke J.N., Simonelli M., Garibaldi M., Ashcroft I., Hague R., Vedani M., Wildman R., Tuck C. (2017). Calorimetric study and microstructure analysis of the order-disorder phase transformation in silicon steel built by SLM. J. Alloys Compd..

[77] Mohamed A.E.-M.A., Zou J., Sheridan R.S., Bongs K., Attallah M.M. (2020). Magnetic shielding promotion via the control of magnetic anisotropy and thermal Post processing in laser powder bed fusion processed NiFeMo-based soft magnet. Addit. Manuf..

[78] Jain V., Kumar P., Bagui S., Halder C., Patra S., Ghosh A. (2023). Comprehensive study on the through-process Goss texture evolution in Fe-3.78 wt.%Si grain oriented electrical steel. Mater. Chem. Phys..

[79] Shen X., Meng F., Lau K.B., Wang P., Lee C.H. (2022). Texture and microstructure characterizations of Fe-3.5wt%Si soft magnetic alloy fabricated via laser powder bed fusion. Mater. Charact..

[80] Riener K., Albrecht N., Ziegelmeier S., Ramakrishnan R., Haferkamp L., Spierings A.B., Leichtfried G.J. (2020). Influence of particle size distribution and morphology on the properties of the powder feedstock as well as of AlSi10Mg parts produced by laser powder bed fusion (LPBF). Addit. Manuf..

[81] Brika S.E., Letenneur M., Dion C.A., Brailovski V. (2020). Influence of particle morphology and size distribution on the powder flowability and laser powder bed fusion manufacturability of Ti-6Al-4V alloy. Addit. Manuf..

[82] Tiismus H., Kallaste A., Rassõlkin A., Vaimann T. (2019). Preliminary Analysis of Soft Magnetic Material Properties for Additive Manufacturing of Electrical Machines. KEM.

[83] Pantělejev L., Koutný D., Paloušek D., Kaiser J. (2017). Mechanical and Microstructural Properties of 2618 Al-Alloy Processed by SLM Remelting Strategy. MSF.

[84] Mazeeva A.K., Staritsyn M.V., Bobyr V.V., Manninen S.A., Kuznetsov P.A., Klimov V.N. (2020). Magnetic properties of Fe–Ni permalloy produced by selective laser melting. J. Alloys Compd..

[85] Kezrane M., Guittoum A., Hemmous M., Lamrani S., Bourzami A., Weber W. (2015). Elaboration, Microstructure, and Magnetic Properties of Nanocrystalline Fe90Ni10 Powders. J. Supercond. Nov. Magn..

[86] Urban N., Masuch M., Paduch J., Franke J. An Approach to Eddy Current Reduction in Laser Powder Bed Fused High Silicon Steel Considering Manufacturing Influences. Proceedings of the 2021 11th International Electric Drives Production Conference (EDPC).

[87] Giraud A., Bernot A., Lefevre Y., Llibre J.F. Measurement of magnetic hysteresis swelling-up with frequency: Impact on iron losses in electric machine sheets. Proceedings of the 2017 IEEE International Workshop of Electronics, Control, Measurement, Signals and their Application to Mechatronics (ECMSM).

[88] Krings A. (2014). Iron Losses in Electrical Machines: Influence of Material Properties, Manufacturing Processes and Inverter Operation.

[89] Goldman A. (2006). Modern Ferrite Technology.

[90] Burkhardt C., Wendler M., Lehnert R., Hauser M., Clausnitzer P., Volkova O., Biermann H., Weidner A. (2023). Fine-grained microstructure without texture obtained by electron beam powder bed fusion for AISI 304 L-based stainless steel. Addit. Manuf..

[91] Koepf J.A., Gotterbarm M.R., Markl M., Körner C. (2018). 3D multi-layer grain structure simulation of powder bed fusion additive manufacturing. Acta Mater..

[92] Liu Z., Tan Z., Yao H., Chen C., Zhou Z., Xue Y., Shao W., Guo X., Yao H., Chen L. (2022). Heat treatment induced microstructural evolution and strength enhancement of Al–Cr–Fe–Ni–V high-entropy alloy fabricated by laser powder bed fusion. Mater. Sci. Eng. A.

[93] Qu S., Ding J., Fu J., Fu M., Song X. (2022). Anisotropic material properties of pure copper with fine-grained microstructure fabricated by laser powder bed fusion process. Addit. Manuf..

[94] Shi T., Li J., Gao G., Sun J., Yang Z., Yan J., Qian G. (2024). Microstructural evolution and formation of fine grains during fatigue crack initiation process of laser powder bed fusion Ni-based superalloy. Addit. Manuf..

[95] Rodriguez-Vargas B.R., Stornelli G., Folgarait P., Ridolfi M.R., Miranda Pérez A.F., Di Schino A. (2023). Recent Advances in Additive Manufacturing of Soft Magnetic Materials: A Review. Materials.

[96] Chen S., Gao H., Zhang Y., Wu Q., Gao Z., Zhou X. (2022). Review on residual stresses in metal additive manufacturing: Formation mechanisms, parameter dependencies, prediction and control approaches. J. Mater. Res. Technol..

[97] Kaess M., Werz M., Weihe S. (2023). Residual Stress Formation Mechanisms in Laser Powder Bed Fusion-A Numerical Evaluation. Materials.

[98] Renuka Balakrishna A., James R.D. (2021). A solution to the permalloy problem—A micromagnetic analysis with magnetostriction. Appl. Phys. Lett..

[99] Zhang B., Fenineche N.-E., Zhu L., Liao H., Coddet C. (2012). Studies of magnetic properties of permalloy (Fe–30%Ni) prepared by SLM technology. J. Magn. Magn. Mater..

